# Should a single growth standard be used to judge the nutritional status of children under age 5 years globally? No

**DOI:** 10.1016/j.ajcnut.2024.04.014

**Published:** 2024-09-10

**Authors:** Harshpal Singh Sachdev, Elaine Borghi

**Affiliations:** 1 Senior Consultant Pediatrics and Clinical Epidemiology, Sitaram Bhartia Institute of Science and Research, New Delhi, India; 2 Department of Nutrition and Food Safety, World Health Organization, Geneva, Switzerland

**Keywords:** growth standards, hunger, overnutrition, stature-for-age, undernutrition, weight-for-age, weight-for-height

## Abstract

Universal growth standards for under-five children, given the worldwide variation in healthy growth and several determinants of anthropometry, are imprecise measures of nutritional status, particularly when used cross-sectionally. In constructing the global-use WHO growth standard, linear growth differences between contributing sites and pooled mean were >0.2 SD in 37% of observations. Systematic reviews confirm even greater variability, notably amplified for weight-for-age and head-circumference-for-age metrics. Unsurprisingly, developed nations had higher, and LMICs lower, growth dimensions. Contextual growth references predict neonatal morbidities, pathological short stature, macrocephaly, cardiometabolic risk factors, and adult noncommunicable diseases better than the WHO standards. Child body composition also varies contextually, with greater adiposity despite comparable weights in South Asian populations. Thus, contextual references, though not the perfect solution, are better suited for everyday practice and nutrition policy. Growth standards should only be used as a screening for clinical judgments aided by precise biomarkers.

## Main Argument (Sachdev)

Conceptually, “under-nutrition” is often conflated with poverty, poor living conditions, agricultural output, or physical strength by stakeholders and the lay public. Nonetheless, a useful definition, among several available, states that “Nutrition refers to the whole process in which the human body takes food from the outside world, and, through digesting, absorbing, metabolizing, and using the beneficial substances, supplies energy, constitutes and updates body tissues, and regulates physiological functions” [1]. Thus, first, nutritional status is principally a biological construct, representing the balance between the nutritional intake (primarily energy, macro- and micro-nutrients) and the nutritional demands of the body.

Second, growth standards for identifying abnormality rely upon marginal cut-offs of anthropometric distributions in populations instead of causal associations with the exposure (nutrition). With significant context-related unevenness in such associations, the comparative test accuracy for risk determination between global and contextual standards may vary across populations.

Third, physical growth refers to temporal anthropometric change. However, growth standards are depicted as attained anthropometry at different ages, largely derived from cross-sectional data. Evaluations of abnormality focus on cross-sectional comparisons (generally below or above 2 SD), especially for public health purposes. But healthy growth is a discontinuous and irregular process with unpredictable spurts followed by periods of stasis [2]. In large data sets, this unevenness averages out; however, a single-time measure in individuals becomes susceptible, creating nuances for clinical applicability. For example, temporary thinness resulting from a linear growth spurt without a corresponding increase in weight can categorize children who were slightly above the global standard cut-off as wasted, but this is unlikely with a lower contextual standard.

Fourth, extensive evidence indicates that body size is determined by several factors [3,4]. These include, among others, genetic; hormonal; nutrition; infections; parental characteristics; socioeconomic status; water, sanitation, and hygiene (WaSH); curative and preventive health care; maternal, household, and community resources; and heat stress. The contribution of nutrition to under-five anthropometry thus varies with context and over time. Undersized children (short, thin, or low-weight) due to nonnutritional causes can, therefore, be nutritionally replete or, paradoxically, even overfed.

Despite the widespread appreciation of these limitations, growth charts are used to judge the nutritional status of under-five children. The Sustainable Development Goal on “Zero Hunger,” too, envisages achieving the internationally agreed targets on stunting and wasting (https://www.undp.org/sustainable-development-goals/zero-hunger) by 2025, based on a universal growth standard.

### Universal growth standard concept

Worldwide variation in human growth has been recognized for ages. There is a continuing debate on whether one universal standard suffices for all or whether different populations should have optimal growth standards. The former has obvious advantages of simplifying comparisons, clinical practice, and policy while sending an impactful message of global equity.

The World Health Organization’s Multicentre Growth Reference Study (WHO-MGRS) charts serve as the global growth standard for under-five children. These emanated from a population-based study in 6 cities in the United States, Oman, Norway, and Brazil, and in selected affluent neighborhoods in Ghana and India [5,6]. It adopted a fundamentally different prescriptive approach by creating growth standards, which reflected how children should grow in all countries rather than the earlier descriptive approach of documenting how they grew at a particular time and place (growth references). They enrolled children who had no apparent socioeconomic, environmental, or biological constraints for growth, were born to nonsmoking mothers, singleton, term births, with no significant disease and followed recommended feeding and health care practices. The longitudinal (birth to 24 months; 208–329/site) and cross-sectional (2–5 years) components enrolled 8440 children. Construction of a single international standard from pooled data was justified because of the “striking similarity in linear growth” between children in the 6 sites. It was concluded that WHO Growth Charts “represent the best description of physiological growth and should be applied to all children everywhere, regardless of ethnicity, socioeconomic status, and type of feeding” and that “a single international standard for assessing the growth of all children embodies the very powerful message that when health and key environmental needs are met, the world’s children grow very similarly” [6].

I will consider potential caveats with this approach, using WHO-MGRS as an example, but the principles are generally applicable.

### Caveats

Notwithstanding the fact that lower (or higher) anthropometric values will identify an undersized (or oversized*)* individual regardless of the cause, the most prevalent nomenclature for undersized children, especially among policy stakeholders, is “undernourished.” Using the WHO-MGRS standard, childhood “undernutrition” in populations is conventionally measured by 3 anthropometric indices: underweight (weight-for-age <−2 SD), stunted (length/height-for-age <−2 SD), and wasted (weight-for-length/height <−2 SD). Whereas a wasted child is considered to have acute undernutrition, stunting represents a longer-lasting nutritional deprivation, and underweight is a composite measure of these two indices. Paradoxically, the WHO-MGRS standards neither quantified biomarkers nor nutrient intakes to accurately determine the nutritional status. Contingent upon the enrolment conditions, these children were simply presumed to be similar, optimally (neither under- nor over-) nourished, and with “strikingly similar” growth curves that were subsequently pooled.

However, this claim of “striking similarity” in growth is questionable. Only stature was evaluated for assessing population differences. Pooling was considered appropriate if differences between the mean and individual sites ranged within ±0.5 SD, which is an arbitrary statistical judgment [6]. With stringent selection criteria and negligible measurement error, linear growth among sites should be nearly identical or, from an interventional perspective, with a maximal window of 0.2 SD, which assumes significance at the population level. In low resource settings, the typical improvement in linear growth with nutritional interventions, if any, is 0.1 SD to 0.2 SD [3,4,7]. Among the urban poor in India, even a composite intervention package of health, nutrition, WaSH, and psychosocial care extending from preconception to early childhood recorded a maximal length gain of 0.46 SD [8]. Thus, a margin of 0.5 SD is too high to judge the similarity of linear growth among selected populations that have no growth constraint. In WHO-MGRS, in 54 comparisons with pooled mean (9 ages at 6 sites), linear growth differences were >0.2 SD in 37% and >0.3 SD in 19% [6]. Further, differences between individual sites, instead of from the pooled mean, are a more accurate indicator of variability between countries. At 2 to 4 years, in comparison to Brazil, the differences ranged from 0.31 to 0.62 SD for India and 0.63 to 0.73 SD for Oman.

Further, any similarity in linear growth does not imply corresponding equivalence in other dimensions, including weight-for-stature, weight-for-age, or head circumference. Absence of relevant published data from WHO-MGRS precludes a direct confirmation or denial of this possibility. However, the INTERGROWTH-21st study evaluated fetal growth and newborn size in 8 geographically defined urban populations in which the health and nutrition needs of mothers were met [9]. Criteria similar to WHO-MGRS, focusing on skeletal dimensions, were used to justify pooling. Notably, Indian infants had lower birthweight (1 SD) and birth length (0.3 SD) from the pooled mean, indicating a lower ponderosity.

The majority of participants screened for the longitudinal and cross-sectional components in Brazil, Ghana, India, and Oman were excluded for socioeconomic reasons [5]. Consequently, the richest, elite population (comprising the upper ∼1%–2% in India) was selected. Unfortunately, numerous children in low- and middle-income countries (LMICs) are still born with growth-hindering factors that are nonmodifiable in the short term, including intergenerational constraint, low birth size, socioeconomic status, and parental literacy. It is unrealistic to expect them to equal the WHO-MGRS growth performance. Thus, apart from setting an aspirational goal that could span over generations, the real-world utility of these standards for the mainstream and underprivileged populations is substantially compromised (see below). Parents want to know how their children are growing given their context (conditional performance) and if improvement is required or possible. Setting unreasonable targets creates the despairing impression, often with knee-jerk remedies, that the achievement of optimal growth is only for affluent and privileged inhabitants!

Apart from limited representation and sample size to adequately capture global variability and other statistical issues [10], the WHO-MGRS growth patterns have not been validated universally. Systematic reviews document patterns of growth that differ from the WHO-MGRS curves, even in socioeconomically advantaged children and with analogous selection criteria [[11], [12], [13]]. In the study, including 55 countries or ethnic groups with 11 million children [11], for linear growth, 20% of the total means were ≥0.5 SD from the MGRS mean, whereas 46% of means diverged by ≥0.25 SD at ≥4 age groups. This variability was amplified for weight-for-age (31% ≥0.5 SD; 62% ≥0.25 SD) and head circumference (54% ≥0.5 SD; 74% ≥0.25 SD). Importantly, variability occurred on both sides. Generally, developed nations had higher, and LMICs lower, growth dimensions than the WHO-MGRS.

Thus, conceptually, the goal of a universal standard is largely elusive, and as stated earlier: “…issues of representativeness, sample inclusion and exclusion, and statistical considerations highlight the important issue that the standard for “normal” growth is highly contingent on the data collected” [10]. Further, with the ongoing secular trend, even in developed countries, such standards could get outdated with time.

### Practical implications

Evidence suggests that the relationship between ill health, including nutrition inadequacy, and body size may be context-specific. As illustrated below, in comparison to contextual references, the WHO-MGRS standards can result in significant misclassification errors, leading to over- or under-diagnosis of malnutrition.

In contrast to anthropometry, metabolic and body composition (adiposity, lean tissue) measures are more proximate, responsive, and reliable predictors of nutritional status, particularly for short-term insults or interventions. Three-compartment model estimates of infant body composition differ among countries despite comparable weights, with lower lean tissue and greater fat mass in Indian infants [14]. Using WHO standards, in an Indian survey, half of the anthropometrically undernourished 5- to 19-year-old children (thin: 54%; stunted: 59%) showed “metabolic obesity” (dysglycemia or dyslipidemia). The upswing in prevalence began at the median of WHO BMI-for-age [15], confirming the reported superiority of contextual references in screening for cardiometabolic risk factors. Also, contextual childhood BMI references proved superior in predicting the risk of developing diabetes and metabolic syndrome in young adulthood [16].

Similarly, contextual references are a better screening tool in routine clinical practice, resulting in better management or avoiding unnecessary diagnostics. Contextual charts were better than INTERGROWTH-21^st^ standards for identifying small-for-gestational-age neonates at risk of adverse outcomes [17]. WHO-MGRS standards tend to overdiagnose macrocephaly and underdiagnose microcephaly in industrialized nations, with the converse possibility in LMICs [11,13]. Substantial misclassification of pathological short stature has been demonstrated in European countries (8%–32%) [18] and other settings. The emerging problem of overweight is overestimated in high-resource countries and underestimated in LMICs. It is therefore not surprising that despite formal endorsement of WHO-MGRS standard by over 125 member states, professional organizations and pediatricians of some countries, including from the United States (2–19 y) [19] and Europe, rely upon national references for everyday practice.

The undesirable consequences of assessing nutritional adequacy from a single growth standard for public health discourse and policy merit priority attention and remedy, especially for the LMICs. The advocacy machinery tends to portray national child undersize statistics as burden of “hunger” or “starvation,” and this is sensationalized by the lay media. The 2023 Global Hunger Index, which uses national stunting and wasting metrics, ranks India at 111 among 125 countries, with a “serious” grading (https://www.globalhungerindex.org). Despite critical flaws in this index, the misdirected shaming creates intense political pressure demanding an inappropriate response for amplification of food or nutrient-product-based supplementation. Ironically, India was a food exporter during the Ukraine war crisis and is currently witnessing a rapid escalation of overnutrition-associated disease and cardiometabolic risk factors, even in children, which will be fueled by misdirected knee-jerk feeding policy responses. This scenario could be substantially restrained and well-directed to those in need, using Indian references, which halve the growth failure burden [20].

### Conclusion

Growth standards, particularly when used cross-sectionally, are imprecise measures of nutritional status. Constructing a universal growth standard appears elusive, especially because of accompanying drawbacks for practical translation, considerably so for some settings, with undesirable consequences. The use of contextual growth references, although admittedly not the perfect solution, will result in a better fit for everyday practice and policy. Growth standards are only a screening tool to reach a clinical judgment, which needs support from other accurate biomarkers to achieve precision in individual and public health nutrition.

## Refutation (Borghi)

In his arguments against the use of global standards for assessing nutrition status, Sachdev conflates the use of global standards for individual assessment with cross-sectional observation at a specific time for the assessment of population nutrition status.

Anthropometry has an important advantage over other nutritional metrics. Biochemical and clinical metrics are useful only at the extremes of malnutrition, whereas body measurements are informative over the full spectrum. This is important for early detection of malnutrition [21]. Moreover, anthropometric metrics provide the best noninvasive indicators of when a child is not reaching their full potential growth, largely but not only due to living in a suboptimal environment for their wellbeing.

It is important to distinguish between the uses of the growth standards for individual assessment and cross-sectional population nutrition status assessment. Stewart et al. [22] define stunted growth as linear growth faltering, which occurs when a child is not growing in length or height in accordance with his or her potential. Individual-level monitoring of children’s growth patterns is important to flag deviations from a child’s expected growth curve in any context. Growth monitoring is the pillar of the promotion of key individual-level interventions, as it helps identify the optimal time to introduce complementary foods, is often used to assess lactation performance, and helps in the diagnosis of growth faltering, failure to thrive, and excessive growth. Despite the small shifts across growth curves among the 6 sites in the Multicentre Growth Reference Study (MGRS), the similarity of the growth patterns was notable, and because the samples of children were based on similar conditions known to be associated with children’s optimal capacity to grow. Those associations are based on extensive evidence, as described by Stewart et al. [22] in their contextual framework for stunted growth and development. Naturally, if conditions are unlike those of the MGRS sample, the growth pattern will differ, not only shifting the size but also ultimately altering the curve, therefore, altering the rate of growth expected for children living in nonoptimal conditions. When children’s growth is compared with contextual, local references, one misses the important growth characteristics of these children compared to the optimal growth depicted by children living under conditions associated with unconstrained growth.

The fixed cut-off for stunting does not necessarily catch, and was not intended to catch, all of the individual children experiencing growth faltering. The cross-sectional use of global standards for stunting and other anthropometric indicators helps identify population deficits, including intergenerational adverse health factors, not only directly related to nutrition adequacy or intake balance but to other important determinants that could be reverted with appropriate interventions at the population level.

In his main argument, Sachdev uses differences among populations to defend the idea that children grow differently in different contexts and that associations with determinants vary. He refers to studies from different countries and ethnic backgrounds following “similar” criteria to the MGRS. It is not possible with cross-sectional samples in such studies to follow such a strict protocol including feeding and socioeconomic criteria.

The work cited by Sachdev on the customization of WHO under-five growth standards [20] exemplifies why contextual references should not be used. The authors of that work apply a “correction” of the WHO growth standards for India, claiming to result in an accurate calculation of the prevalence of stunting, underweight, and wasting in Indian children based on a cross-sectional sample of children (NFHS-5 data), attempting to estimate growth faltering without longitudinal data. They estimate lower levels of malnutrition compared to when using the standard definitions of malnutrition based on the WHO child growth standards. Another attempt to underestimate malnutrition was depicted in work by Subramanian et al. [23] where an adjusted metric for stunting was to be used for monitoring and evaluating nutrition policies in India. As we previously noted, the adoption of such a method, intentionally minimizing the growth deficits, will substantially disincentivize the commitment and actions that are urgently needed to improve the inadequate living conditions of hundreds of millions of children globally [24].

## Rebuttal (Sachdev)

Borghi’s assertion of conflating the use of global standards for individual and population nutrition status assessment is perplexing. When assessing child growth [25], the diagnostic nomenclature for abnormal anthropometry (e.g., wasted or obese) does not vary for clinical practice or population surveys. Thus, a severely wasted child and a population with a high prevalence of severely wasted children will both trigger actions to address severe acute malnutrition at either the individual or population level. Longitudinal growth records are undoubtedly ideal for early detection of growth faltering and for a better etiological diagnosis, but these are seldom available in many LMICs, and the practitioner has to decide on a single cross-sectional measurement. In any case, the perceived conflating, if any, does not differentially inform the selection of global or contextual standards.

The contention that anthropometry is informative over the full spectrum of malnutrition, especially for its early detection, is not supported by directly relevant evidence. This “important advantage” is selectively cited from a commentary [21], which also states: “The main disadvantage of anthropometry is its lack of specificity, as changes in body measurements are also sensitive to several other factors, including intake of essential nutrients, infection, altitude, stress and genetic background.” Also, from a biological perspective, decreases (or increases) in nutritional intakes will first result in metabolic changes to which the body could adapt; if not, consequences like growth faltering (or excess) will begin to appear. Thus, in the current era of rapid technological advances, anthropometry cannot be presumed to be a superior diagnostic test to precise biomarkers for evaluating nutritional status. Reviews on the effects of overfeeding [26] and energy restriction [27] in humans confirm that excess energy intake results in metabolic findings of dysglycemia and dyslipidemia. As pointed out earlier, paradoxically, in Indian children, there is frequent intraindividual co-existence of anthropometrically defined undernutrition and these overnutrition-associated biomarkers [15]; are these individuals undernourished or overnourished? Finally, micronutrient deficiencies, even for evaluating the public health burden, are evaluated through biomarkers instead of anthropometric deficits.

The WHO-MGRS growth standards from 2−5 years were based on cross-sectional data only. Thus, it is unscientific to dismiss a large body of global evidence [[11], [12], [13]] solely on cross-sectional design or even with less-rigorous protocols when the magnitude and direction of potential measurement biases have not been ascertained. Further, both these considerations are not applicable to the observed differences in newborn anthropometry within the 8 country sites in the INTERGROWTH-21st study [9].

This debate sets up the distinctions between science and the politics of science in establishing nutritional standards. Borghi’s concluding statement indicates her inclination for the latter: “…the adoption of such a method, intentionally minimizing the growth deficits, will substantially disincentivize the commitment and actions…of hundreds of millions of children.” Two phrases deserve attention. “*Intentionally minimizing*” attributes ulterior motives that do not exist. But the other phrase, “*disincentivizing the commitment*” is telling. Fear of lowering the assessed undernutrition burden cannot be a consideration for defending and propagating a single growth standard, tilting public policy-making toward a Procrustean bed.

## Acknowledgments

HSS is grateful to Prof. Anura V. Kurpad for his constructive feedback on earlier drafts of this manuscript.

## Author contributions

The authors’ responsibilities were as follows – HSS created the main text and rebuttal; EB created the refutation; and all authors read and approved the final manuscript.

### Conflicts of interest

The authors report no conflicts of interest.

### Funding

The authors reported no funding received for this study.

### Disclaimer

This article series is designed as an Oxford-style debate. As such, participants are required to argue pro- and con- positions, even when that opinion may differ from their own. The views expressed in this debate do not necessarily reflect the opinion of the participants, *The American Journal of Clinical Nutrition*, or the American Society for Nutrition.
